# 120. Antimicrobial Prescribing Guidance and Communication Among Health Care Professionals in Five Guatemalan Hospitals

**DOI:** 10.1093/ofid/ofab466.322

**Published:** 2021-12-04

**Authors:** Brooke M Ramay, Clara I Secaira, Nuria Chavez, Mario Augusto Melgar Toledo, Randall M Lou-Meda, Nancy V Sandoval, Herberth G Maldonado

**Affiliations:** 1 Universidad del Valle de Guatemala, Center for Health Studies, Paul G. Allen School for Global Health, Washington State University, Pullman, USA, Guatemala City, Sacatepequez, Guatemala; 2 Centro de Estudios en Salud, Guatemala, Solola, Guatemala; 3 Hospital Regional de Zacapa, Guatemala, Zacapa, Guatemala; 4 Hospital Roosevelt, Guatemala City, Totonicapan, Guatemala; 5 FUNDANIER, Guatemala, Sacatepequez, Guatemala; 6 Unidad de Cirugía Cardiovascular de Guatemala, Guatemala, Quetzaltenango, Guatemala

## Abstract

**Background:**

Communication among health care professionals during antimicrobial prescribing is critical to ensure appropriate use. This is of concern in Guatemala where physicians seldom consider guidance from other professionals during antimicrobial prescribing activities.

**Methods:**

We carried out a cross sectional questionnaire and open ended interviews with physicians from five hospitals in Guatemala to describe perceptions of communication between health care providers, and acceptance of antimicrobial guidance during prescribing.

**Results:**

From January to April 2021 an electronic questionnaire was sent to enrolled physicians of which 74% completed participation (n=107/145). Fifty-five percent participated in open ended interviews (n=79/145). Respondents perceived high levels of communication between physicians and ID specialists (94% of respondents); 52%, and 54% perceived high levels of physician-pharmacist, and physician-nurse communication respectively. Significant differences in the perception of physician-pharmacist communication were detected when comparing responses between hospitals, and between respondent sex (chi^2^, p< 0.05). Barriers to communication between professionals included lack of local guidelines or protocols, patient overload, COVID-19 pandemic, lack of mentorship, and little room to discuss antimicrobial therapy with higher-ranking physicians. Eighty percent and 45% of physicians were open to receiving antibiotic optimization recommendations from other physicians, and pharmacists respectively. Notable barriers to accepting recommendations from pharmacists included lack of regular communication, lack of clinical experience, and concern about evidence based recommendations.

**Conclusion:**

Effective communication is perceived between physicians during antimicrobial prescribing activities. Marginal levels of communication and acceptance of prescribing recommendations have been detected between physicians and pharmacists.In this milieu, there is an opportunity to strengthen multidisciplinary teams to optimize antimicrobial use.

**Disclosures:**

**Mario Augusto Melgar Toledo, MD**, **Merck** (Grant/Research Support)**Pfizer** (Grant/Research Support)

